# Evaluating Entomopathogenic Nematodes as Biocontrol Agents Against Two Major Cockroach Species, *Blattella germanica* and *Periplaneta americana*, in Antalya, Türkiye

**DOI:** 10.3390/pathogens14070655

**Published:** 2025-07-01

**Authors:** Aysegul Cengiz, Burak Polat, Sevval Kahraman Kokten, Ummuhan Aslan Bıckı, Cansu Calıskan, Samed Koc, Emre Oz, Serap Kocaoglu-Cenkci, Ozge Tufan-Cetin, Huseyin Cetin

**Affiliations:** 1 Department of Biology, Faculty of Science, Akdeniz University, 07070 Antalya, Türkiye; aysegulcengiz@akdeniz.edu.tr (A.C.); bpolatant@gmail.com (B.P.); sevvaldilarakahraman@gmail.com (S.K.K.); cansucaliskan0724@gmail.com (C.C.); 2 Department of Forest Protection, Wildlife and Protected Areas, Southwest Anatolia Forest Research Institute, 07010 Antalya, Türkiye; ummuhanaslan@ogm.gov.tr; 3 Laboratory Animals Application and Research Centre, Akdeniz University, 07070 Antalya, Türkiye; samedkoc@akdeniz.edu.tr; 4 Department of Medical Services and Techniques, Vocational School of Health Services, Antalya Bilim University, 07190 Antalya, Türkiye; emre.oz@antalya.edu.tr; 5 Department of Nutrition and Dietetics, Faculty of Health Sciences, Akdeniz University, 07070 Antalya, Türkiye; skocaoglu@akdeniz.edu.tr; 6 Department of Environmental Protection Technology, Vocational School of Technical Sciences, Akdeniz University, 07070 Antalya, Türkiye; ozgetufan@akdeniz.edu.tr

**Keywords:** biological control, *Blattella germanica*, entomopathogenic nematodes, *Periplaneta americana*, pest management

## Abstract

Cockroaches, particularly the German cockroach (*Blattella germanica* Linnaeus, Blattodea: Ectobiidae) and the American cockroach (*Periplaneta americana* (Linnaeus), Blattodea: Blattidae), are major public health pests due to their ability to transmit pathogens and develop resistance to chemical insecticides, including synthetic pyrethroids, which are widely used worldwide. Given the increasing resistance, entomopathogenic nematodes (EPNs) have emerged as a potential biological control alternative. This study evaluates the efficacy of three EPN species, *Steinernema carpocapsae* (Weiser), *S. feltiae* (Filipjev), and *Heterorhabditis bacteriophora* Poinar, against *B. germanica* and *P. americana* collected from different regions of Antalya, Türkiye. Laboratory bioassays were conducted under controlled conditions, testing five EPN concentrations (100, 250, 500, 750, and 1000 IJs/mL). The results showed that *S. carpocapsae* was the most effective, causing mortality rates of 46.7% to 100% in adult German cockroaches and 20% to 66.7% in nymphs, while *S. feltiae* and *H. bacteriophora* exhibited lower efficacy. American cockroaches showed higher resistance, with *S. carpocapsae* achieving a maximum mortality of 33.3% at the highest concentration, whereas *S. feltiae* and *H. bacteriophora* had no significant lethal effect. These findings suggest that *S. carpocapsae* could be a promising biological control agent for *B. germanica*, particularly in pyrethroid-resistant populations.

## 1. Introduction

Cockroaches are represented globally by approximately 4600 species, with only about 30 of these species inhabiting environments shared with humans. Among these species, the German cockroach (*Blattella germanica* Linnaeus, Blattodea: Ectobiidae) and the American cockroach (*Periplaneta americana* (Linnaeus), Blattodea: Blattidae) are the most significant due to their close association with human environments [1,2,3]. The German cockroach is characterized by its high reproductive capacity, with females producing up to five oothecae during their lifetime, each containing approximately 30–40 embryos. Previous studies have demonstrated that protein-rich diets can significantly enhance reproductive output and population growth in cockroaches by accelerating ootheca production and shortening developmental cycles [4]. Its life cycle can be completed in as little as 2 to 3 months under favorable environmental conditions, although it may extend up to 100 days depending on temperature and nutrition. German cockroaches prefer warm, dry environments and are commonly found in enclosed areas with a steady food source, such as kitchens, warehouses, restaurants, hospitals, and food businesses [5]. In contrast, the American cockroach, which is significantly larger than the German cockroach, thrives in cooler and more humid environments. Female *P. americana* can produce 6 to 14 oothecae during their lifespan, with each ootheca containing 14–16 embryos. The development time of this species is longer, typically taking 6 to 12 months to complete a generation depending on environmental conditions. American cockroaches are often found in sewers, basements, and other damp areas, where they seek shelter and reproduce [6].

The German cockroach (*B. germanica*) is known for its ability to transmit a variety of pathogens, posing significant public health risks [7,8]. This cockroach can carry and spread bacteria such as *Salmonella enterica* serovar *typhimurium* and shiga toxin-producing *Escherichia coli*, which are responsible for intestinal infections [9]. Additionally, it can transmit viral agents like norovirus and sapovirus, which play a key role in foodborne outbreaks. Research suggests that this cockroach functions not only as a mechanical but also as a biological vector, with genetic variations potentially influencing its effectiveness in transmitting pathogens. It is also noted for its ability to carry and spread parasites such as *Toxocara canis* Werner. Furthermore, its fecal matter contains allergenic proteins, including immunoglobulin E-reactive proteases, which can trigger severe allergic reactions in susceptible individuals [10,11]. Similarly, the American cockroach (*P. americana*) is a well-documented vector of pathogens that threaten human health. This species has been implicated in the mechanical transmission of bacteria, including *Staphylococcus aureus*, *Klebsiella pneumoniae*, and *Pseudomonas aeruginosa*, as well as fungi such as *Aspergillus* and *Candida* [12]. It is also capable of spreading intestinal parasites like *Entamoeba histolytica* and *Giardia lamblia* [13]. Like the German cockroach, its feces, shed skins, and saliva are significant sources of allergens that can exacerbate asthma and other allergic conditions, especially in urban environments. The larger size and mobility of the American cockroach enhance its ability to contaminate a wide range of surfaces, making it an important pest in food storage and preparation areas [14].

In the fight against cockroach infestations, biocidal products in the form of sprays, dust, baits, or gels are commonly used to control both cockroaches [15]. These products contain synthetic pyrethroids (deltamethrin, alphacypermethrin, lambda-cyhalothrin, and cypermethrin), organophosphate insecticides (chlorpyrifos, malathion), phenylpyrazole insecticides (fipronil), and neonicotinoid insecticides (dinotefuran). While these products are generally effective in managing *B. germanica* and *P. americana* in many areas, research indicates that both species have developed resistance to various groups of insecticides. For instance, resistance to pyrethroids has reached up to 100-fold in some populations of German cockroaches [16], and resistance to phenylpyrazole insecticides like fipronil has been observed, with some populations showing resistance levels increasing by 36-fold [17]. Similarly, studies on *P. americana* populations have reported resistance to pyrethroids, organophosphates, and phenylpyrazoles, with significant variability depending on regional insecticide use patterns. In Türkiye, a study conducted by Oz et al. [18] identified high resistance levels to synthetic pyrethroids (deltamethrin, alphacypermethrin, lambda-cyhalothrin) in *B. germanica*, with resistance ratios exceeding 545-fold in some populations. The primary causes of this resistance in both species include enhanced metabolic detoxification of these chemicals and mutations in their target sites [19]. Resistance development in both *B. germanica* and *P. americana* underscores the importance of integrated pest management (IPM) approaches, including rotation of insecticide classes, non-chemical control methods, and improved sanitation practices, to effectively combat these pests. In addition to the loss of efficacy and the development of resistance to chemicals used in the fight against cockroaches, the application of these biocides in environments where humans and animals live has negative effects on both the environment and human health. The use of these chemicals in enclosed spaces can pose significant risks to human health. Insecticides such as pyrethroids and organophosphates can cause health issues such as respiratory problems, skin irritation, allergic reactions, and neurotoxic effects when people are exposed to them for extended periods [20]. Furthermore, the chemical residues left behind after the use of these biocides can lower indoor air quality and may contaminate food and water sources, posing long-term risks to human health. Studies have shown that exposure to these residues, particularly among vulnerable groups such as children, can be linked to behavioral problems and reproductive health issues [21]. This is especially dangerous for vulnerable groups, such as children, the elderly, and individuals with chronic conditions. The continuous accumulation of chemical residues increases the potential for adverse impacts on public health.

As mentioned above, due to the public health importance of cockroaches, their increasing resistance to chemical products, and the negative effects of these chemicals on both the environment and human health, researchers have shifted their focus toward safer, more environmentally friendly, and effective biological control methods. One promising approach is the use of EPNs, recognized as potent biological control agents [22]. These nematodes, comprising over 120 species from the families *Steinernematidae* and *Heterorhabditidae*, belong to the genera *Heterorhabditis*, *Steinernema*, and *Neosteinernema* within the order Rhabditida. These small roundworms, which have been developed into biopesticides, act as obligate parasites that invade and kill insects by reproducing within their tissues. Found on every continent except Antarctica, many species have been isolated from soils in agricultural and natural ecosystems [23]. Their broad host range, environmental safety, and compatibility with integrated pest management practices make them highly effective agents for biological control [24].

Considering all research findings, this study, conducted for the first time in Türkiye, investigates the lethal effects of three entomopathogenic nematode species [*Steinernema carpocapsae* (Weiser) (SC), *S. feltiae* (Filipjev) (SF), and *Heterorhabditis bacteriophora* Poinar (HB)], which were obtained as commercially available biopreparations with well-documented virulence against a wide range of insect pests. Their infectivity has been previously demonstrated in laboratory bioassays against *Spodoptera littoralis* (Boisduval), *Leptinotarsa decemlineata* Say, and *Alphitobius diaperinus* Panzer [25,26,27]. In the present study, these nematodes were tested at various application rates against the nymphs and adults of two medically important cockroach species: the German cockroach (*B. germanica*) and the American cockroach (*P. americana*). Six *B. germanica* and four *P. americana* populations collected from different locations in Antalya, Türkiye, were evaluated. This research provides valuable insights into the potential use of EPNs as biological control agents against insecticide-resistant cockroach populations.

## 2. Materials and Methods

### 2.1. Tested Cockroaches

In this study, American cockroaches (*P. americana*) were collected from sewer systems in four regions of Antalya (Teomanpaşa, Arapsuyu, Pınarbaşı, and Uncalı) four years ago and reared under laboratory conditions. German cockroaches (*B. germanica*) were collected in 2018 from six locations (Ahatlı, Dokuma, Güllük, Gürsu, Lara, and Şirinyalı), including establishments such as restaurants and bakeries. Both species were collected in numbers ranging from 50 to 100 individuals to establish laboratory colonies. German cockroaches, previously confirmed to be resistant to synthetic pyrethroids (alpha-cypermethrin, deltamethrin, lambda-cyhalothrin, and permethrin) based on our earlier study, were maintained in 5 L cylindrical plastic containers, whereas the American cockroaches, which did not exhibit significant resistance, were housed in 15 L rectangular plastic boxes [18]. Both colonies were reared under controlled conditions (25 ± 2 °C, 60 ± 5% relative humidity, and a 12:12 light–dark photoperiod) at the Vector Ecology and Control Laboratory of Akdeniz University. The cockroaches were provided with ad libitum access to a mixture of cornmeal, starch, and honey, along with water. Rolled cardboard pieces were placed in the containers to cater to their preference for dark environments, and the rearing setup was cleaned every 3–4 weeks to ensure optimal hygiene. Mixed-gender adult cockroaches were used in each replicate, with at least two individuals from each sex to ensure gender balance. Adults were 2 weeks old, and nymphs were 1 month old at the time of testing, selected based on physiological maturity.

### 2.2. Tested Nematodes

In this research, three entomopathogenic nematode (EPN) species—*Steinernema carpocapsae* (Ekobioset^®^), *S. feltiae* (Nematac^®^), and *Heterorhabditis bacteriophora* (Bioteam^®^)—were examined. These were obtained as commercial biopreparations produced by Bio-Global Zirai Biyolojik Sistemler Tar. Dan. Gid. Tar. San. ve Tic. A.Ş. (Bahçelievler Mah. Konyaaltı Cad. No: 54/13 Muratpaşa, Antalya, Türkiye). Each product consisted of approximately 50 million infective juveniles (IJs) in the third larval stage, formulated in a moist, gel-like matrix resembling soft agar. To ensure maximum viability, we first prepared the cockroach populations and then ordered the nematode products. Upon arrival, the products were stored at 4 °C and used within 3–7 days, which is well within the typical one-month shelf life recommended for such formulations under refrigeration. Before application, the nematodes were diluted in distilled water to prepare working suspensions at concentrations ranging from 100 to 1000 IJs/mL.

### 2.3. Nematode Efficacy Testing on Cockroaches

In this study, biological efficacy tests were conducted using nymphs and adults of both German (*B. germanica*) and American (*P. americana*) cockroaches, with adults consisting of mixed-gender groups. For German cockroaches, individuals were placed in sterile Petri dishes (63.5 cm^2^ surface area × 1.5 cm height) containing ten cockroaches each (either nymphs or adults) and exposed to varying rates of infective juveniles (IJs/mL) of three nematode species in distilled water. Similarly, American cockroaches were tested in plastic containers (113 cm^2^ surface area × 7 cm height) with flat bases, where filter paper was placed on the bottom, and nematodes were applied in the same manner. For American cockroaches, five individuals were used per replicate. Although only five *P. americana* individuals were used per replicate, this number was selected based on preliminary trials indicating that higher densities in confined arenas caused elevated stress and increased physical interactions among individuals. As *P. americana* is a significantly larger and more active species than *B. germanica*, lower density helped to ensure more stable behavioral conditions and reliable mortality assessments. This approach has also been supported by previous studies evaluating large-bodied insects under similar laboratory conditions.

One milliliter of the solutions containing 100, 250, 500, 750, or 1000 infective juveniles (IJs)/mL was applied to the bottoms of the Petri dishes and containers. The concentrations of 100, 250, 500, 750, and 1000 IJs/mL were selected based on preliminary laboratory screening and align with the range commonly used in previous bioassays evaluating the efficacy of EPNs against insect pests [25,28]. This range allowed for detecting a dose-dependent response while remaining within practical application limits. For *B. germanica*, each Petri dish contained 10 individuals, resulting in approximately 10, 25, 50, 75, or 100 IJs per cockroach. For *P. americana*, each container housed 5 individuals, resulting in approximately 20, 50, 100, 150, or 200 IJs per cockroach. Test arenas were checked on days 3, 6, 9, and 12 to record mortality. Additionally, 1 mL of distilled water was added at each interval to maintain moisture on the filter paper and preserve nematode viability. To meet the cockroaches’ water requirements, a piece of moist cotton (~1 cm in size) was placed in each container. Mortality was assessed by counting the individuals at each time point, and control groups received only distilled water. As a food source, pellet-form fish feed was provided to the cockroaches on the same days. Experiments were conducted under controlled conditions (24 ± 2 °C, 50 ± 10% relative humidity, and a 12:12 light photo period), and each treatment, including controls, was replicated three times. Cockroaches were considered dead if they exhibited no movement after being prodded for 1 s three times, and the presence of nematodes in dead cockroaches was confirmed by dissection on the final day of the experiments. To confirm nematode infection, all cockroach cadavers were dissected under a stereomicroscope using fine-tipped forceps. Internal tissues, especially from the abdominal cavity, were carefully extracted and examined for the presence of infective juveniles (IJs) or developing nematodes under both stereomicroscope and compound microscope at 100–400× magnification. The dissection and confirmation procedures were conducted following two protocols [28,29].

### 2.4. Statistical Analysis

Mortality data were adjusted using Abbott’s formula when the control mortality ranged between 5% and 20%; no adjustment was made if the control mortality was below 5% [30]. Differences among the groups were analyzed through one-way ANOVA, and Duncan’s new multiple range test was applied to identify specific groups contributing to significant differences. The independent samples *t*-test was used to determine whether there was statistical evidence that two associated population means were significantly different. All statistical analyses were conducted with SPSS version 20.

## 3. Results

In this study, the infectivity of three entomopathogenic nematode species was investigated on German cockroach (*B. germanica*) populations collected from six regions and American cockroach (*P. americana*) populations collected from four regions in Antalya Province, Türkiye. According to the results, SC caused significantly higher mortality in German cockroaches after 12 days compared to the other two nematode species (SC were found significantly different for nymphs *F*: 150,584 and for adults *F*: 390,889; *df*: 1293, *p* ≤ 0.000), while it led to low mortality (≤33.3%) in American cockroach populations even at the highest concentration. In contrast, *S. feltiae* and *H. bacteriophora* showed almost no virulence on American cockroach nymphs and adults at the tested concentrations.

After 12 days, none of the nematodes exhibited a significant lethal effect on either nymphs or adults of the American cockroach. Only SC caused average mortality rates of 20% and 33.3% in nymphs at the highest concentration (1000 IJs/mL) in the Teomanpaşa and Pınarbaşı populations, respectively. At all other tested concentrations, the remaining nematodes showed no discernible lethal activity on either adults or nymphs of American cockroaches, with results not significantly different from those in the control group. In the tests conducted with American cockroaches, mortality in the control groups did not exceed 5% in either nymphs or adults by the end of the 12th day. As no significant mortality was observed in American cockroaches at the tested nematode concentrations compared to the control group, this section includes only a general explanation, and near-zero data were not visualized in graphs.

**Figure 1:**
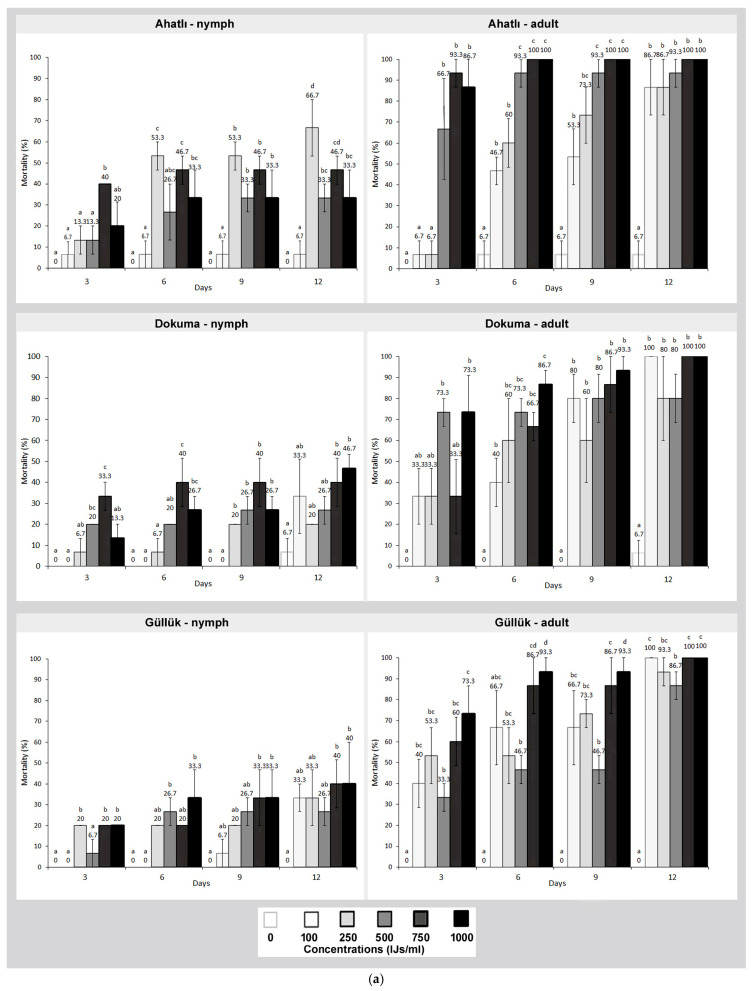

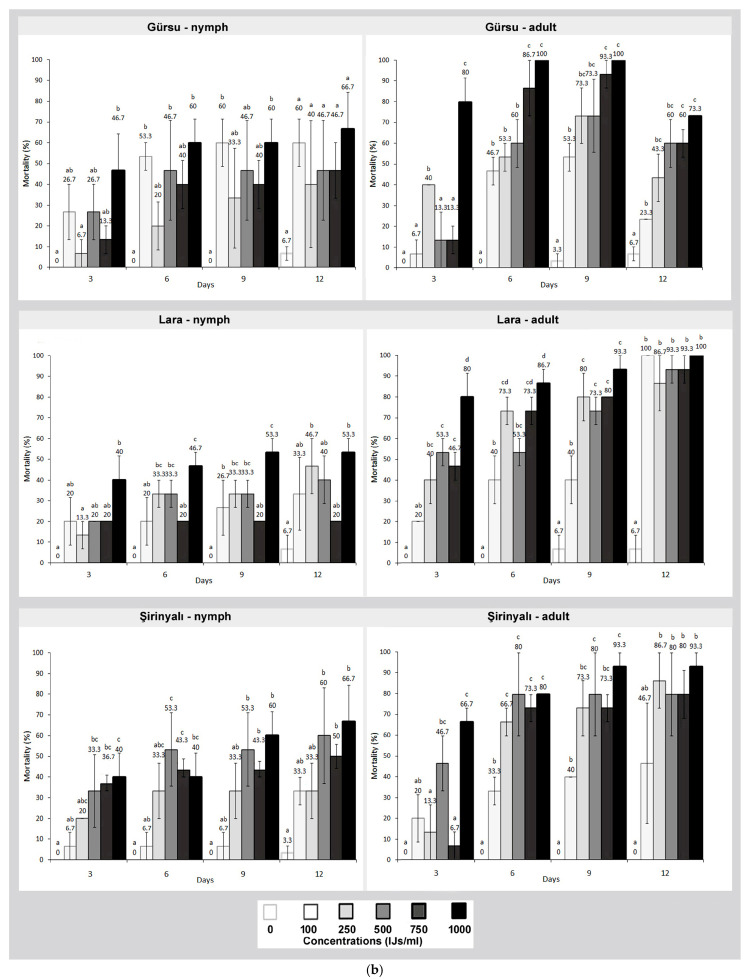
(**a**) Lethal effect of the entomopathogenic nematode *Steinernema carpocapsae* on nymphs and adults of three German cockroach (*Blattella germanica*) populations from Ahatlı, Dokuma, and Güllük at different concentrations (100–1000 IJs/mL) and time intervals (3–12 days). Different lowercase letters on the bars indicate statistically significant differences between concentrations within the same day (Duncan multiple range test, *p* ≤ 0.05). (**b**) Lethal effect of the entomopathogenic nematode *Steinernema carpocapsae* on nymphs and adults of three German cockroach (*Blattella germanica*) populations from Gürsu, Lara, and Şirinyalı at different concentrations (100–1000 IJs/mL) and time intervals (3–12 days). Different lowercase letters on the bars indicate statistically significant differences between concentrations within the same day (Duncan multiple range test, *p* ≤ 0.05).

The tested nematodes exhibited a notable lethal effect on German cockroaches, depending on the nematode species, tested concentrations and the life stage of the cockroach. When the effect of SC nematode on German cockroaches was examined, nymphs were found to be more resistant than adults (*F*: 125,799; *df*: 862, *p* ≤ 0.000). At the tested concentrations, the lethal effect on adults varied between 46.7% and 100% by the end of the 12th day. In the Dokuma, Lara, Güllük, and Ahatlı populations, mortality rates exceeding 80% were observed at concentrations of 500 IJs/mL and above. When the mortality rates of nymphs were examined, the average mortality rate varied between 20% and 66.7% by the end of the 12th day (Figure 1a,b).

**Figure 2:**
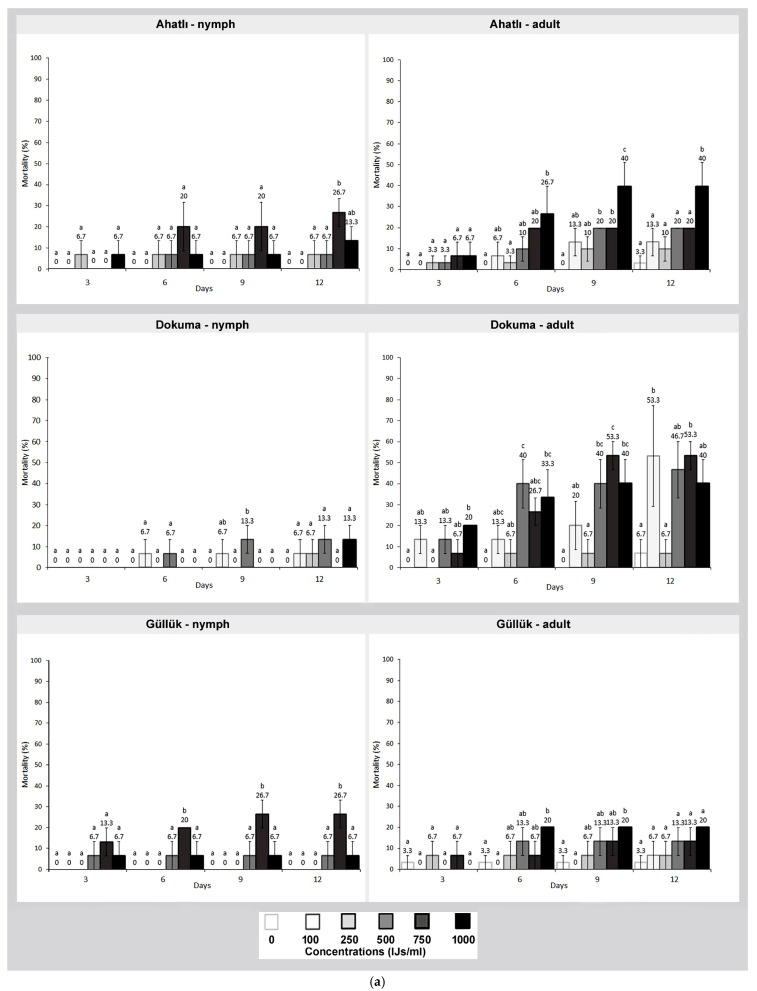

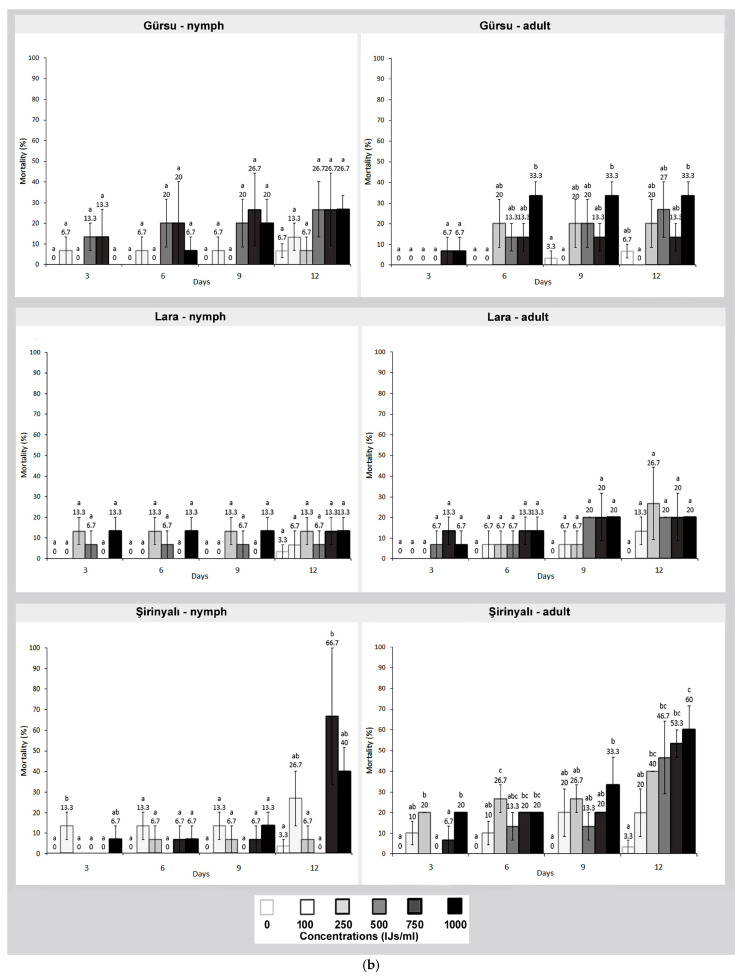
(**a**) Lethal effect of the entomopathogenic nematode *Steinernema feltiae* on nymphs and adults of three German cockroach (*Blattella germanica*) populations from Ahatlı, Dokuma, and Güllük at different concentrations (100–1000 IJs/mL) and time intervals (3–12 days). Different lowercase letters on the bars indicate statistically significant differences between concentrations within the same day (Duncan multiple range test, *p* ≤ 0.05). (**b**) Lethal effect of the entomopathogenic nematode *Steinernema feltiae* on nymphs and adults of three German cockroach (*Blattella germanica*) populations from Gürsu, Lara, and Şirinyalı at different concentrations (100–1000 IJs/mL) and time intervals (3–12 days). Different lowercase letters on the bars indicate statistically significant differences between concentrations within the same day (Duncan multiple range test, *p* ≤ 0.05).

When the effect of SF nematode on German cockroaches was examined, the lethal effect on adults varied between 0% and 53.3% at the tested concentrations by the end of the 12th day. In the Dokuma and Şirinyalı populations, mortality rates exceeding 40% were observed at concentrations of 500 IJs/mL and above. When the mortality rates of nymphs were examined, the average mortality rate varied between 0% and 26.7% by the end of the 12th day (Figure 2a,b). Although mortality rates were quite low in both nymphs and adults, the mortality rate of adults was statistically higher than that of nymphs (*F*: 27,194; *df*: 862, *p* ≤ 0.000).

**Figure 3:**
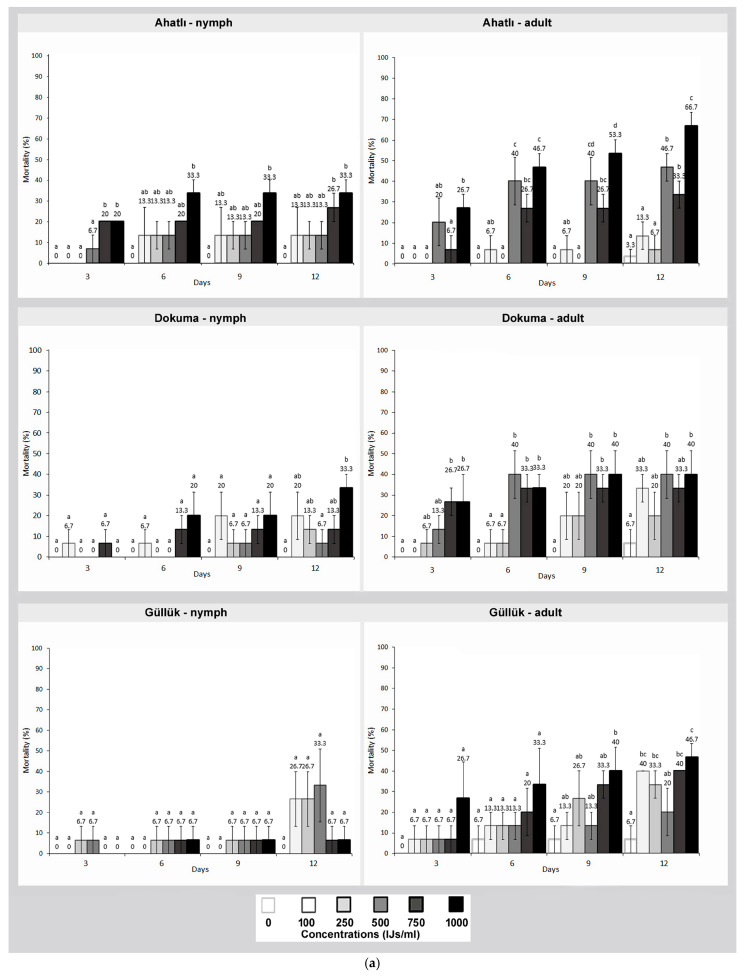

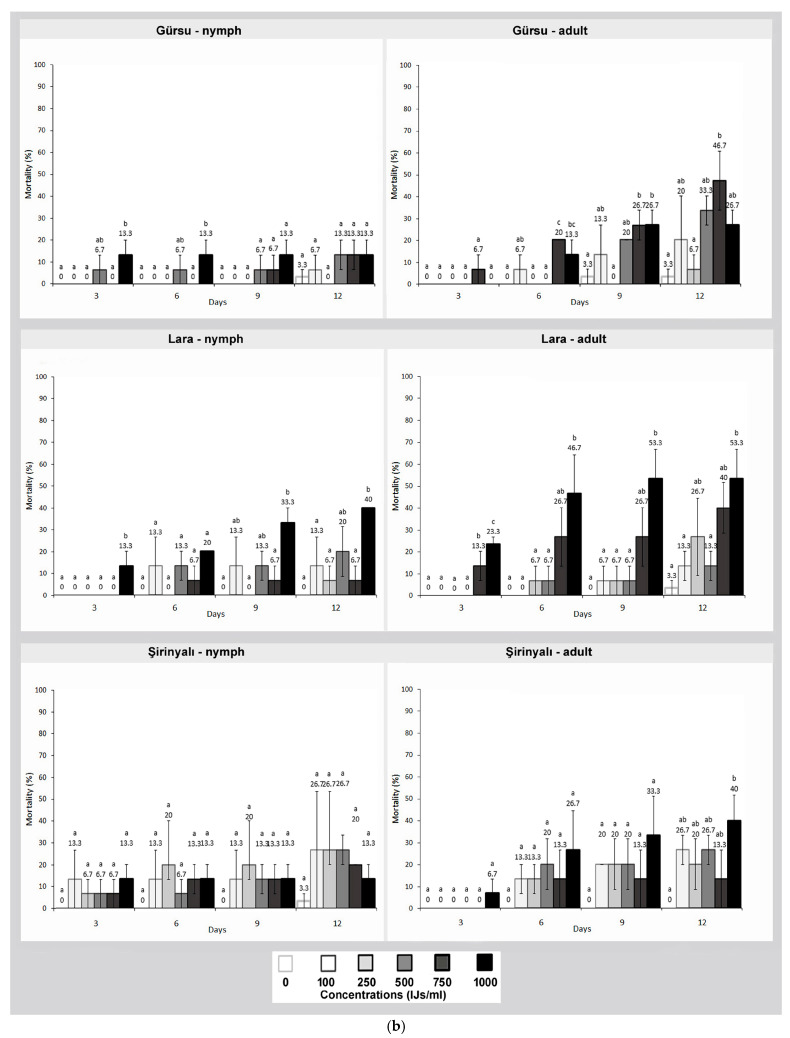
(**a**) Lethal effect of the entomopathogenic nematode *Heterorhabditis bacteriophora* on nymphs and adults of three German cockroach (*Blattella germanica*) populations from Ahatlı, Dokuma, and Güllük at different concentrations (100–1000 IJs/mL) and time intervals (3–12 days). Different lowercase letters on the bars indicate statistically significant differences between concentrations within the same day (Duncan multiple range test, *p* ≤ 0.05). (**b**) Lethal effect of the entomopathogenic nematode *Heterorhabditis bacteriophora* on nymphs and adults of three German cockroach (*Blattella germanica*) populations from Gürsu, Lara, and Şirinyalı at different concentrations (100–1000 IJs/mL) and time intervals (3–12 days). Different lowercase letters on the bars indicate statistically significant differences between concentrations within the same day (Duncan multiple range test, *p* ≤ 0.05).

When the effect of HB nematode on German cockroaches was examined, nymphs were found to be more resistant than adults (*F*: 46,516; *df*: 862, *p* ≤ 0.000). At the tested concentrations, the lethal effect on adults varied between 6.7% and 66.7% by the end of the 12th day. In nymphs, the average mortality rates ranged between 6.7% and 40% (Figure 3a,b).

When evaluating the lethal effect of nematodes among the tested German cockroach populations, no significant differences were observed among populations. However, the overall lethality values of each nematode varied. In this context, SC was the most effective nematode against German cockroaches, followed by HB and SF, respectively (for nymphs *F*: 150,584 and for adults *F*: 390,889; *df*: 1293, *p* ≤ 0.000) (Figure 1 and Figure 2). In the control groups of German cockroaches, an average mortality rate between 0% and 6.7% was recorded by the end of the 12th day.

## 4. Discussion

In our literature reviews, it was found that various EPNs, isolated by different researchers from soil or deceased insects, or commercially produced and sold in bulk, exhibited varying levels of lethal effects on cockroaches. These effects varied depending on the cockroach species, life stage (nymph, adult) gender (male or female), application method (e.g., bait, spray), abiotic conditions of the environment (e.g., temperature, humidity), and the tested dose/concentration.

Appel et al. [31] conducted laboratory and field evaluations of SC for controlling *B. germanica*. Laboratory tests showed that nematode-treated stations achieved a lethal time (LT_50_) of 2.06–12.64 days, with higher doses leading to faster mortality. Choice box experiments revealed repellency effects, which decreased at higher nematode concentrations, and a performance index combining mortality and repellency indicated that stations with 2 × 10^6^ nematodes had the greatest efficacy. Field trials in infested apartments demonstrated significant reductions in cockroach populations, comparable to traditional insecticide baits, with peak effectiveness observed eight weeks after treatment. In our study, particularly in our observations on the German cockroach, we found that nematode-induced mortality in adult cockroaches began on day 3, depending on the nematode species, concentration, and the cockroach’s life stage, reaching nearly 90% on days 6 and 9 (for SC *F*: 26,888, for SF *F*: 27,600, for HB: 31,521; *df*: 860, *p* ≤ 0.000). This is likely due to the time required for young nematodes to complete their development after entering the cockroach’s body and subsequently causing harm to the host.

Morton and García-del-Pino [28] reported that the susceptibility of young adult males and females of two cockroach species, *P. americana* and *Capnodis tenebrionis* L., to the SC was tested at doses of 50 and 100 IJs/cm^2^. Their findings revealed that males were significantly more susceptible than females, with mortality rates exceeding 97% in males compared to less than 58% in females under similar conditions at 14 days post-infection. The study also demonstrated that nematodes primarily entered the insects through common routes such as the mouth and cloaca, but male-specific entry via the genital aperture was significant, a route not observed in females. This difference was attributed to anatomical or physiological barriers in females that limited nematode penetration. These results highlight the importance of considering sex-related differences in pest control strategies and suggest that targeting males could enhance the efficacy of SC as a biological control agent. In our research, we found that SC had a notably high lethal effect on adult German cockroaches, with all tested concentration levels producing mortality rates ranging from 40% to 100%, depending on the population. However, because we did not distinguish between sexes when selecting adults for the tests and therefore did not track which sex died first, we were unable to draw any specific conclusions regarding sex-based differences in mortality. At the same time, the concentration we used against American cockroaches was lower than that used by them and since we did not differentiate adult insects by sex, no comparison could be made.

El-Kady et al. [32] investigated the pathogenicity of SC on *B. germanica* using filter paper and bait assays, with doses ranging from 25 to 1000 IJs per five adults. They found that mortality rates increased with higher doses and longer exposure times. The filter paper assay showed higher virulence (LD_50_: 85.64 IJs) compared to the bait assay (LD_50_: 106.94 IJs). Similarly, based on the data from our research, as the SC application concentration and exposure time increased, mortality rates also rose. In another study, El-Kady et al. [29] compared bait applications (banana and cat food) and spray applications for controlling German cockroach populations. They reported that spraying SC in the environment was more effective than bait application.

The efficacy of bait formulations composed of cat food and attapulgite clay, supplemented with various concentrations of a local *Steinernema* sp. and an imported SC strain, was assessed by Maketon et al. [33] against American cockroaches (*P. americana*) and German cockroaches (*B. germanica*). They reported that SC was highly effective against German cockroaches, achieving 86.7 ± 4.7% mortality, whereas *Steinernema* sp. caused 57.7 ± 8.0% mortality. In contrast, both nematode strains showed limited effectiveness against American cockroaches. In another study investigating the effects of different nematode species on various cockroach species, Cutler et al. [34] examined the susceptibility of three cockroach species [*Gromphadorhina portentosa* (Schaum), *Nauphoeta cinerea* (Olivier), and *Blaptica dubia* (Serville)] to EPNs. The study tested *Steinernema kraussei* and a combination of *Steinernema* spp. and *Heterorhabditis* spp. at doses of 50 and 150 nematodes/cm^2^. *S. kraussei* was found to be harmless to all species, with no significant impact on survival or feeding. In contrast, *B. dubia* was highly susceptible to the nematode mixture, showing significant mortality within six days of exposure, along with reduced feeding. Additionally, the nematodes successfully reproduced in the cadavers of *B. dubia*.

An investigation by Baker et al. focused on assessing how temperature variations (15 °C, 20 °C, and 25 °C) influence the reproduction and pathogenicity of SC and HB in *B. germanica* [35]. They reported that SC was more virulent than HB at all tested temperatures and exposure times. At 20 °C, SC caused 100% mortality in nymphs within 48 h, while at 25 °C, it achieved the same result in adults after 72 h. Reproduction rates of SC were significantly higher at 25 °C, whereas HB exhibited poor reproductive outcomes under all conditions. The researchers concluded that SC is more effective against *B. germanica*, particularly at 20–25 °C, making it more suitable for use in spring and autumn. The results of our laboratory tests at 24 ± 2 °C support these findings, showing that SC is more effective than HB.

A recent study evaluated the susceptibility of the American cockroach to two isolates of *Steinernema rarum* (OLI and N105 strains) collected from Córdoba, Argentina [36]. Two doses of nematodes (3200 and 6400 IJs/host) were tested at 25 °C. The OLI isolate caused significantly higher mortality rates (67% and 89% for the lower and higher doses, respectively) compared to the N105 isolate, which did not exceed 65% mortality. Both isolates completed their life cycle in *P. americana*, with OLI producing a larger number of IJs per host. As a result of our study, numerous IJs were detected in the cadavers of German cockroaches that were killed by the three tested nematode species.

The limited number of studies in the literature on testing nematodes against cockroaches may be due to various factors. One challenge is the long developmental period of cockroaches, as German cockroaches take more than three months to reach adulthood, while American cockroaches can take nearly a year. Additionally, maintaining cockroach colonies, ensuring consistent infection rates, and the need for specialized laboratory conditions could further complicate research in this area. The primary focus of pest control studies has traditionally been on chemical insecticides, limiting the exploration of biological control methods such as EPNs. Another factor could be the shorter shelf life of nematode-based formulations compared to chemical insecticides, which may affect their commercial viability. The data obtained in this study indicate that nematodes like SC can make a significant contribution to resistance management. Its high efficacy against all six synthetic pyrethroid-resistant German cockroach populations tested in a study is a major advantage. Expanding research in this area by testing different nematode species and optimizing application methods could enhance the potential of nematodes in integrated cockroach management strategies [37].

American cockroaches demonstrate higher resistance to EPNs compared to German cockroaches at the concentrations tested in our research. This resistance is likely due to factors such as thicker exoskeleton, higher body position relative to the ground, and potential evolutionary adaptations resulting from exposure to parasitic nematodes commonly found in fecal matter [38,39]. This observation aligns with findings from other researchers, who have also reported greater resistance to EPNs in American cockroaches. Interestingly, German cockroach nymphs have been shown to exhibit higher resistance to EPNs compared to adults, mirroring their increased resistance to insecticides. Studies indicate that late-stage nymphs possess higher LC_50_ and LD_50_ values for various insecticides (e.g., bendiocarb, chlorpyrifos, permethrin and cypermethrin), attributed to factors such as thicker cuticles, behavioral avoidance, and enhanced detoxification mechanisms mediated by enzymes like esterases and glutathione S-transferases [40,41]. Moreover, our findings suggest that incorporating EPNs into integrated control programs may help manage resistance in *B. germanica* populations. However, further field studies are necessary to evaluate whether these nematodes exhibit comparable infectivity and persistence in complex urban environments. Additionally, nematodes that develop within the carcasses of dead cockroaches could potentially spread to other individuals through cannibalism, thus extending their effect across generations. Although *B. germanica* prefers dry environments, it often inhabits moist microhabitats such as behind kitchen appliances, near drains, or in basements. These areas may retain sufficient humidity for EPNs to remain viable long enough to establish contact. Additionally, formulating EPNs into gel-based or viscous spray formulations similar to those used in cockroach baits may improve persistence and contact efficiency in such microhabitats. However, further research is needed to confirm the feasibility and long-term impact of this approach under field conditions. Although mixed-gender adults were used based on preliminary trials that showed no significant sex-based differences in susceptibility, potential effects of gender on EPN efficacy should still be considered in future studies.

## 5. Conclusions

This study highlights the potential of EPNs as biological control agents against German and American cockroaches. Among the tested nematode species, SC demonstrated the highest efficacy against German cockroaches, with mortality rates reaching 100% in some populations. However, American cockroaches exhibited greater resistance to all tested nematodes, with mortality rates remaining significantly lower. These findings suggest that EPNs, particularly SC, could be integrated into pest management strategies for German cockroaches, especially in insecticide-resistant populations. Further studies are needed to optimize application methods, explore the potential of different nematode strains, and assess field efficacy under varying environmental conditions. The integration of EPNs into IPM programs could offer an environmentally friendly and sustainable alternative to chemical insecticides, reducing public health risks associated with cockroach infestations.

## Data Availability

The data presented in this study are available within the article’s figures; additional information can be obtained from the corresponding author upon reasonable request.

## Abbreviations

The following abbreviations are used in this manuscript:

±	Plus–Minus
%	Percent
~	Tilde (Approximately)
≤	Less than or Equal to
^®^	Registered Trademark Symbol
ANOVA	Analysis of Variance
cm	Centimeters
cm^2^	Square Centimeters
°C	Degree Celsius
*df*	Degrees of freedom
e.g.,	Exempli Gratia (For Example)
EPNs	Entomopathogenic Nematodes
*F*	F distribution
HB	*Heterorhabditis bacteriophora*
LC_50_	Lethal Concentration
LD_50_	Lethal Dose
LT_50_	Lethal Time
IJs	Infective Juveniles
IPM	Integrated Pest Management
mL	Milliliters
nth	Ordinal Numbers
N105	N105 strain of *Steinernema rarum*
OLI	OLI strain of *Steinernema rarum*
*p*	Probability
SPSS	Statistical Package for the Social Sciences
SC	*Steinernema carpocapsae*
SF	*Steinernema feltiae*
